# Relationship of serum adiponectin and resistin to glucose intolerance and fat topography in south-Asians

**DOI:** 10.1186/1475-2840-5-10

**Published:** 2006-05-02

**Authors:** Hanif Wasim, Nasser M Al-Daghri, Raja Chetty, Phillip G McTernan, A H Barnett, Sudhesh Kumar

**Affiliations:** 1 Queen Elizabeth Hospital, Birmingham University, Birmingham, UK; 2 King Saud University College of Science Biochemistry Department, Riyadh, Saudi Arabia; 3 Birmingham Heartland Hospital, Medicine Department, Birmingham B9 5SS, UK; 4 University of Warwick, Warwick Medical School, Diabetes and Metabolism Unit, Coventry, CV4 7AL, UK; 5 Department of Medicine, Division of Medical Sciences, Birmingham University, UK; 6 Heartlands Hospital, Edgbaston Birmingham B15 2TH, UK

## Abstract

**Objectives:**

South-Asians have lower adiponectin levels compared to Caucasians. It was not clear however, if this intrinsic feature is related to aspects of glucose metabolism. This study aims to determine the relationship between body fat distribution and adipocytokine in South-Asian subjects by measuring serum adipocytokines, adiposity, insulinemia, and glucose tolerance levels.

**Methods:**

In this cross-sectional study, 150 South-Asians (80 males, 70 females) were included, 60 had NGT (Control group, Age 51.33 ± 11.5, BMI 27 ± 2.3), 60 had IGT (Age 57.7 ± 12.5, BMI 27.2 ± 2.7), 30 had type 2 DM (Age 49.5 ± 10.9, BMI 28 ± 1.7). Measures of adiposity, adipocytokines and other metabolic parameters were determined. Parameters were measured using the following: a) Plasma glucose by glucose oxidase method b) CRP by immunoturbidimetric method (Roche/Hitachi analyser) c) insulin by Medgenix INS-ELISA immunoenzymetric assay by Biosource (Belgium) d) Leptin, Adiponectin by radioimmunoassay kits by Linco Research (St. Charles MO) e) Resistin by immunoassay kits by Phoenix Pharmaceuticals INC (530 Harbor Boulevard, Belmont CA 94002, USA).

**Results:**

Adiponectin concentrations were highest in NGT, decreased in IGT and lowest in DMT2, (both p < 0.01). Leptin was significantly higher in DMT2 than IGT and NGT p = 0.02 and 0.04 respectively. There was a significant positive relationships between log adiponectin and 2-hr insulin values, p = 0.028 and history of hypertensions and a ischemic heart disease p = 0.008 with R = 0.65. There was a significant inverse correlation between log adiponectin and resistin, p < 0.01.

**Conclusion:**

Resistin levels had an inverse correlation with adiponectin levels, indicating an inverse relationship between pro-inflammatory cytokines and adiponectin. Adiponectin levels were related to glucose tolerance.

## Introduction

The association between excess adipose tissue and the development of type 2 diabetes, dyslipidaemia and cardiovascular disease has long been recognised. It is now established that adipose tissue is a significant endocrine organ of humans that secretes over 30 proteins many of which have actions on the processes of fat accumulation and metabolism. Numerous studies have attempted to identify several mechanisms linking obesity with insulin resistance and type 2 diabetes, and factors have been suggested to play in obesity-related insulin resistance such as tumour necrosis factor-α(TNF-α), interleukin-6 (IL-6), non-esterified fatty acids (NEFA), resistin and adiponectin [1]. Adiponectin, the gene product of the adipose most abundant gene transcript 1 (apM1) [2], is a collagen-like protein that is exclusively synthesized in white adipose tissue and has been postulated to have an important role in the modulation of glucose and lipid metabolism in insulin-sensitive tissues both in humans and animals. Circulating adiponectin concentrations are decreased in obese [3] and type 2 diabetes [4] and the reduction is believed to have a role in the pathogenesis of cardiovascular diseases associated with obesity and other components of the metabolic syndrome [5-7]. Recent evidence has also suggested the role of adiponectin in the regulation of insulin action, energy homeostasis, obesity and insulin resistance. Circulating adiponectin levels and adiponectin gene expression in adipose tissue are reduced in patients with type 2 diabetes [8-10]. Available data suggest that adiponectin might reduce hepatic glucose production and increase muscle glucose utilisation, perhaps by increasing fat oxidation and thereby reducing circulating NEFA levels and intramyocellular accumulation [11]. Studies of adipocytokines in populations with different propensity for obesity, insulin resistance, type 2 diabetes and atherosclerosis are needed and South-Asians are interesting in this respect, because they have very high incidence of insulin resistance, central obesity, type 2 diabetes and cardiovascular disease [12-17]. In a previous study by Valsmakis et al [18], South-Asians have lower adiponectin levels compared to Caucasians. It was not clear however, if this feature of the South-Asians is related to aspects of glucose metabolism. This study aims to determine the relationship between body fat distribution and adipocytokines in South-Asian subjects by measuring serum adiponectin, resistin, and leptin, measures of adiposity, insulinemia and glucose tolerance levels.

## Subjects and methods

In this cross sectional study, 150 subjects (80 males, 70 females), all South-Asians were studied, the patients were matched for BMI, and there were 60 subjects with NGT (Non-diabetic control group), 60 subjects with IGT and 30 with DM. The ethics approval was taken from East Birmingham Hospital local ethical committee. The patients were recruited from community gatherings and were invited to the hospital.

### Clinical assessment

This included height, weight, body mass index, waist and hip circumference and sagittal diameter. The Holtain Khan abdominal caliper by Holtain Ltd. (Crymych, UK) was used to measure sagittal abdominal diameter. Each patient was examined supine on a firm examination table. Using sliding callipers with parallel blades a direct reading can be made between its lower arm (touching the subjects back) and its sliding upper arm (touching the front of the subjects abdomen). Tanita fat monitoring machine by Tanita UK Ltd. (Yiewsley, Middlesex UB7 7RY, UK)was used to measure body fat percentage and lean body mass, with the patient standing bare footed on the machine, which calculated these parameters.

### Laboratory measurements and analytical procedures

The patients were requested to come to the hospital fasting and had baseline bloods done with the serum saved for analysis. They had a standard 2 hr oral glucose tolerance test done along with the measurements of fasting and 2 hr insulin. Plasma glucose was measured using glucose oxidase method, while high sensitive C-reactive protein was measured using immunturbidimetric method (Roche/Hitachi analyser). Insulin was determined using the Medgenix-INS-ELISA immunoenzymetric assay by Biosource-Europe S.A. (B-1400 Nivelles, Belgium) [19]. Serum leptin and adiponectin were measured using the radio-immunoassay (RIA) commercially available kit by Linco Research (St Charles MO-63304-USA) [20]. Serum resistin was measured using the enzyme immunoassay kit by Phoenix pharmaceuticals INC (530 Harbor Boulevard, Belmont, CA 94002, USA [21]. Human resistin sensitivity is 4 ng/ml with interassay and between assays variability of <14% and <5% respectively.

### Statistical analysis

Data were analysed using the Statistical Package for the Social Sciences (SPSS for Windows, version 10). Data are expressed as mean (standard deviation) or as median and range if not normally distributed. Leptin and adiponectin values were logarithmically transformed to normalise the data. Groups were compared by student's unpaired t-test or one-way ANOVA was used for inter-group comparisons with normally distributed data and if not normally distributed, by the Mann Whitney U test. Simple and partial correlation coefficients between the variables were determined and multiple regression analysis was done to determine relationships between variables of interest.

## Results

A total of 150 subjects all South-Asians were studied, (80 males,70 females), 60 subjects had normal glucose tolerance, 60 had impaired glucose tolerance and 30 had newly diagnosed type 2 diabetes. The baseline characteristics are shown in table 1. The BMI matched subjects in the three groups, were similar in anthropometric measurements and fat topography. There was no significant difference with respect to percentage body fat, waist circumference, hip circumference and sagittal diameter. There was a statistically significant difference observed with respect to 2-hr insulin level, which was highest in the IGT group and lowest in patients with NGT, a reflection of the change in beta cell function across the glycaemic spectrum. Also CRP and HOMIR were higher in DM patient than NGT p = 0.02 and 0.04 respectively. Adiponectin concentrations were highest in subjects with NGT but decreased in subjects with IGT and lowest in type 2 diabetics, (both p < 0.01. (Fig 1). Leptin concentration was significantly higher in DM than IGT and NGT p = 0.02 and 0.04 respectively. We examined the relationship of log adiponectin with other adipocytokines cross the spectrum of glycaemia, and also other parameters presented in Table 1 like insulin resistance and inflammatory markers like CRP. When adiponectin was used as a dependent variable there was a significant positive relationships with 2-hr insulin values, p = 0.028 and history of hypertensions and ischemic heart disease p = 0.008 with R = 0.65. There was a significant inverse correlation between log adiponectin and resistin, p < 0.01.

**Table 1:** Physical and metabolic characteristics of the study population (mean ± SD). NGT IGT DM

	NGT	IGT	DM
Age	51.33 ± 11.5	57.7 ± 12.5*	49.5 ± 10.9
BMI (kg/m 2)	27 ± 2.3	27.2 ± 2.7	28 ± 1.7
Percent body fat	26.4 ± 4.9	27.2 ± 5.6	22.8 ± 2.3
Waist Circumference (cm)	96.6 ± 18.1	101 ± 9.4	102.4 ± 8.2
Hip Circumference (cm)	100.8 ± 17.9	103.6 ± 7.4	106.8 ± 7.1
Sagittal Diameter (cm)	24.4 ± 3.0	24.1 ± 3.8	25.5 ± 2.7
C-reactive protein #	3.5(0.4–18)	4.1(0.4–17)	6.1(0.8–13)*1
Adiponectin (μg/ml) #	18.7(1.5–65.6)	15.2(2.5–81.6)*	12.2(1.2–37.5)*1
Resistin (ng/ml) #	2.9(1.3–2.7)	3.7(1.4–6.1)	3.2(1.5–12.5)
Leptin (ng/ml) #	2.2(1.7–46.7)	10.1(2.8–34.3)	14.5(3.7–39.6)*2
Fasting insulin (pmol/L)	14 ± 9.7	16.8 ± 23.3	15.1 ± 6.5
2-hr Insulin (pmol/L)	72.1 ± 53	132.6 ± 54.5**	97.4 ± 78.7**
Fasting Glucose (mmol/L)	5.1(0.7)	5.7(1.1)	7.9(2.3)
30 min glucosa (mmol/L)	8.5 (1.5)	10.1 (1.5)	12.3 (1.7) **
2 h glucosa (mmol/L)	5.8 (1.1)	9.3 (1.6)	15.1 (3.7) **
HOMIR#	4.8 (1.1–40.1)	3.8 (1.3–24.5)	4.9 (1.9–8.7) *2

*p = 0.03, *p = 0.022, *p = 0.04 ** p < 0.001 compared to the NGT. All data presented in mean (SD) except # at median (Range)

We also compared subjects with NGT, IGT and type2 diabetes to see the effect of change in glucose tolerance on adiponectin levels, and what effect if any this has on other adipocytokines. The adiponectin levels decreased across the glycaemic spectrum from NGT to type 2 diabetes. There was a significant decrease in the levels of adiponectin between the NGT and IGT subjects, p < 0.05, similarly there were differences between NGT and DM subjects, p < 0.05. The decrease between IGT and DM was not statistically significant. The resistin levels increased across the glycaemic levels, the results were statistically significant between NGT and IGT subjects, p < 0.05 though this difference was not statistically significant between NGT and DM subjects.

## Discussion

In a previous study it was shown that adiponectin concentrations were significantly lower in the South-Asians when compared to Caucasians [18]. In recent study, subjects with type 2 diabetes and impaired glucose tolerance test showed significantly decreased serum adiponectin concentrations. Although serum adiponectin levels were negatively correlated with BMI, diabetic subjects had lower values of serum adiponectin than did non-diabetic subjects, independent of the BMI [8,22,23]. Weyer et al, showed that serum adiponectin concentrations were more closely related to fasting insulinemia and to the rate of insulin-stimulated glucose disposal, a direct measure of insulin sensitivity, than to percent body fat and the 2-hr glucose concentration suggesting that hyperinsulinaemia and/or insulin resistance might be a major determinants of the hypoadiponectinemia in obesity and type 2 diabetes. One of possible mechanisms for this that has been suggested is, overproduction of TNF-αby adipose tissue [24-26]. Also in the recent study showed AdipoR1/R2 appears to be inversely regulated by insulin in physiological and pathophysiological states such as fasting/refeeding, insulin deficiency, and hyper-insulinemia models via the insulin/phosphoinositide 3-kinase/Foxo1 pathway and is correlated with adiponectin sensitivity[27]. Adiponectin interferes with TNF-α signalling in endothelial cells [28]. Decreased serum adiponectin may play a causative role in the development of insulin resistance.

In the present study in BMI matched South-Asian subjects, we found that adiponectin concentration decrease across the glycaemic spectrum, with NGT subjects having the highest and subjects with type 2 diabetes the lowest concentrations. There is a significant decrease in the adiponectin concentrations even at the stage of IGT. In a different study it was found that serum adiponectin concentrations are lower in nondiabetic Pima Indians than in Caucasians and in subjects with IGT and diabetes compared with those with NGT, this indicates that factors other than adiposity must play a role in determining adiponectinemia [9]. In our study serum adiponectin levels were negatively correlated with 2-hr plasma insulin concentration, though there was no relation with percentage body fat. Circulating adiponectin levels have been shown to decrease in parallel with progression of insulin resistance during development of type 2 diabetes in rhesus monkeys genetically predisposed to develop insulin resistance [29]. In this study, there was a negative correlation of adiponectin levels with body weight and fasting insulin levels and a positive correlation with insulin-stimulated glucose up-take (a marker of insulin sensitivity). In these monkeys, the decline in adiponectin levels preceded overt hyperglycaemia. Development of hyperinsulinaemia is one possible mechanism for the suppression of adiponectin levels seen in this study. However, hyperinsulinaemia per se seems unlikely as a mediator of low adiponectin levels, since adiponectin levels remain low in the later stage of type 2 diabetes in association with decreased insulin levels. Adipocyte insulin action or signal transduction rather than absolute levels of insulin may regulate adiponectin concentration. In support of this contention, Bogan and Lodish [30] have shown that secretion of adiponectin by 3T3-L1 adipocytes requires phospatidylinositol 3-kinase (PI-3K), a major intermediate of insulin signalling activity. Insulin stimulated insulin receptor substrate 1 (IRS-1) associated PI-3K activity has been shown to be decreased in adipocytes of type 2 diabetic subjects [31]. Thus it is possible that the decreased adipocyte PI-3K activity in type 2 diabetic patients may contribute to the decreased adiponectin levels. Other investigators have presented data on the potential inverse relationship between adiponectin and insulin action. Euglycemic hyperinsulinaemia clamp studies both in human and rats [32] have shown that insulin infusion leads to decreased circulating adiponectin levels, consistent with the interpretation that insulin exerts an acute effect on adipocytes to decrease production and/or secretion of this adipocytokines. Also some studies showed a relation between the adiponectin and Coronary heart disease risk factors, these results collectively indicate that plasma HDL cholesterol levels and visceral fat masses are independently associated with plasma adiponectin concentrations[33]. In another study a reduction in serum adiponectin level is associated with the prevalence and magnitude of systemic atherosclerosis including IHD and ASO[34] which confirmed our finding in the South Asian subjects.

In our study there was a significant increase in the resistin levels between NGT and IGT subjects though there was no statistically significant difference between NGT and DM subjects. The negative correlation between serum resistin and adiponectin levels may also suggest that, resistin could be a marker for TNF-α which has been shown to be associated with chronic inflammation and insulin resistance. Previous studies have provided evidence for the association of leptin with key variables of the metabolic syndrome and with insulin sensitivity, independently of obesity [35]. Several recent studies have documented a relationship between leptin and insulin levels or insulin sensitivity [36-38]. However, given the close correlation of leptin with indices of adipose tissue mass, no studies were able to investigate if leptin relates to those variables through obesity and/or hyperinsulinaemia, or independent of them. In our study we could not elucidate any relationship between leptin levels according to glucose tolerance.

Taken together, our findings indicate that the observed changes in adiponectin are likely to be related to metabolic changes associated with progression to diabetes. In this study in a British South-Asian population with a high incidence of type 2 diabetes, cardiovascular disease, central obesity and metabolic syndrome, we have demonstrated that serum adiponectin levels are inversely correlated across the glycaemic spectrum with patients of type 2 diabetes having the lowest levels. Even in patients with IGT the serum adiponectin levels were lower than BMI matched NGT subjects. Adiponectin concentration was also negatively correlated with 2-hr plasma insulin levels; these are consistent with the interpretation that insulin exerts an acute effect on adipocytes to decrease production and/or secretion of this adipocytokines.
